# Hydrogen Storage in Pure and Boron-Substituted Nanoporous Carbons—Numerical and Experimental Perspective

**DOI:** 10.3390/nano11092173

**Published:** 2021-08-25

**Authors:** Lucyna Firlej, Bogdan Kuchta, Katarzyna Walczak, Catherine Journet

**Affiliations:** 1 Laboratoire Charles Coulomb, University of Montpellier-CNRS, 34095 Montpellier, France; kasia.walczak1991@gmail.com; 2 Department of Physics and Astronomy, University of Missouri, Columbia, MO 65211, USA; 3 Laboratoire Madirel, University Aix Marseille-CNRS, 13396 Marseille, France; bogdan.kuchta@univ-amu.fr; 4 Department of Micro, Nano and Bioprocess Engineering, Faculty of Chemistry, Wroclaw University of Science and Technology, 50370 Wroclaw, Poland; 5 Laboratoire des Multimatériaux et Interfaces, University Claude Bernard-CNRS, 69622 Lyon, France; catherine.gautier@univ-lyon1.fr

**Keywords:** hydrogen storage, nanoporous carbons, numerical simulations of adsorption

## Abstract

Nanoporous carbons remain the most promising candidates for effective hydrogen storage by physisorption in currently foreseen hydrogen-based scenarios of the world’s energy future. An optimal sorbent meeting the current technological requirement has not been developed yet. Here we first review the storage limitations of currently available nanoporous carbons, then we discuss possible ways to improve their storage performance. We focus on two fundamental parameters determining the storage (the surface accessible for adsorption and hydrogen adsorption energy). We define numerically the values nanoporous carbons have to show to satisfy mobile application requirements at pressures lower than 120 bar. Possible necessary modifications of the topology and chemical compositions of carbon nanostructures are proposed and discussed. We indicate that pore wall fragmentation (nano-size graphene scaffolds) is a partial solution only, and chemical modifications of the carbon pore walls are required. The positive effects (and their limits) of the carbon substitutions by B and Be atoms are described. The experimental ‘proof of concept’ of the proposed strategies is also presented. We show that boron substituted nanoporous carbons prepared by a simple arc-discharge technique show a hydrogen adsorption energy twice as high as their pure carbon analogs. These preliminary results justify the continuation of the joint experimental and numerical research effort in this field.

## 1. Introduction

In the face of progressive depletion of fossil fuels, increasing pollution, and global warming, hydrogen has been widely considered as a major element of our energy future. Today the benefits of being able to produce hydrogen in a ‘clean’ way (using renewable energy sources), and of its clean combustion (to water) have started to counterbalance the substantial industrial difficulties resulting from hydrogen’s low volumetric energy density. In this context, the development of efficient hydrogen storage at low energy expense has become an urgent need. Physisorption in a suitable adsorbent constitutes an attractive alternative to currently used compression and liquefaction, especially for mobile applications. For the automotive sector, the US Department of Energy has fixed a set of technological targets that an adsorption-based storage system should meet to be successfully applied at large scale [1]. However, the appropriate porous material, fulfilling at room temperature even the most basic storage requirement of ~4.5 wt% of adsorbed hydrogen (0.45 kg H_2_/kg system, 28 kg H_2_/m^3^), has not yet been developed.

Carbon-based adsorbents are the most frequently studied and remain very promising hydrogen sorbents considering their availability, low weight, and low cost. Over the last three decades, the early euphoric reports of over 60 wt% hydrogen storage in carbon nanofibers at room temperature and relatively low pressure of ~110 bars [2] were (realistically) scaled down to ~1.5–2 wt%. This low storage capacity is a consequence of weak attraction between hydrogen and carbon (low heat of hydrogen physisorption on carbon-based materials, ~5 kJ/mol); on the other hand, this weak physical interaction between sorbent and hydrogen guarantees process reversibility, essential for effective application in real devices. Similarly, the surface accessible for adsorption of standard activated carbons, of the order of 2000–3000 m^2^/g, is too small, but techniques to increase it have been well identified.

In this paper we summarize our 15-year effort to design model nanoporous carbon with structural and energetic characteristics optimized to attain hydrogen storage objectives formulated for automotive applications. Starting from numerical predictions of storage limits in idealized activated carbons [3], we analyze the possibilities of increasing their surface accessible for adsorption [4,5,6,7,8,9], and of increasing the strength of hydrogen interaction with adsorbents by adequate chemical modification of the carbon pore walls [10,11,12,13,14]. We show that, in any realistic design of efficient hydrogen sorbent, the interplay between structural (accessible surface) and energetic parameters (energy of adsorption) determining capacity of a sorbent must be taken into account [15]. We finish our analysis with experimental validation of numerical predictions, and we present the first experimental evidence that boron substituted carbons, synthetized using the arc-discharge between boron containing graphite electrodes, exhibit hydrogen adsorption energy twice as high as their unsubstituted analogs.

## 2. Numerical Models and Computational Details

The local structure of activated carbons depends critically on the carbon-containing precursor and the preparation procedure. Modeling of such complex structures is challenging, as it has to support a large variety of material parameters, such as density, homogeneity, structure flexibility, pore size distribution, distribution of adsorption sites with a given energy of adsorption, and many others. Many numerical models of activated carbons, considering different sets of material characteristics, have been proposed in the literature [16]. As the experimental data show that locally their structure contains randomly oriented graphene fragments that form slit shaped pores of nanometric size (especially in nanoporous carbons with specific surface area (SSA) larger than 2000 m^2^/g) [7,16], we focused our analysis of hydrogen adsorption on this type of structure. The slit-shaped pore model is very well adapted to modeling of adsorption in porous materials: if the pore walls are structureless, any particular pore is characterized by its width only. In addition, if we assume that the pore extends to infinity (the wall surface is infinite) the adsorption at the pore edges can be ignored. Such a model is a ‘zero-order’ approximation of any real system, and can be easily modified to analyze the influence of pore wall dimension, shape, and chemical heterogeneity on the absorptive properties of the material.

The models of H_2_–H_2_ interaction have been extensively described in the literature [17,18]. As practical applications require hydrogen adsorption at room temperature (T = 298 K), and material characterization is usually performed at liquid nitrogen boiling point (T = 77 K), both temperatures are sufficiently high to considered H_2_ molecules as structureless super-atoms [18] interacting via Lennard-Jones potential: ε_H2–H2_ = 34 K, σ_H2–H2_ = 0.296 nm. The interaction parameters for C–C contacts were set at ε_C–C_ = 28.4 K and σ_C–C_ = 0.334 nm [17,19,20]. In the pure carbon adsorbents, the atomic corrugation of the graphene wall was not explicitly treated, as it represents less than 5% of the H_2_–wall interaction energy, and its influence on the total storage capacity is small. Lorentz-Berthelot mixing rules were applied to determine the values of parameters for heterogeneous H_2_–C contacts. In all interaction models, we have included the temperature-dependent quantum Feymann-Hibbs correction that makes the effective energy of adsorption weaker, and the size of H_2_ super-atom larger [21,22,23]. Although these variations have negligible consequences on the simulated adsorption at room temperature, at T = 77 K the correction of interaction parameters is of the order of 10% and substantially modifies the numerical estimations of the adsorbed amount [3].

In the case of chemically modified surfaces, a fraction of carbon atoms was substituted by heteroatoms (boron, beryllium, or beryllium dimer). Ab initio calculations were then performed to determine the distribution of hydrogen binding energy as a function of both: the distance of the H_2_ molecule from the surface and its location over the substituted surface. The ab initio studies were performed applying the second order Moller-Plesset level of theory using the restricted open Hartree-Fock wave function. The numerical details have been given in the original papers [13,14]. The ab initio parameters were then used to parametrize an effective Lennard-Jones potential describing H_2_–wall interaction [10,11,12,13,14]. The 3D grids of energy distribution in the pores were then calculated, for various B and Be substitution ratios and patterns and different pore widths. These grids were implemented in a home-made Grand Canonical Monte Carlo (GCMC) code. 

The simulations were carried out at T = 77 K and T = 298 K, at a range of pressures between 0 and 120 bars. All Monte Carlo runs were carefully equilibrated (~10^7^ MC steps/molecule in production runs, preceded by long stabilization runs, ~10^6^ MC steps/molecule). All calculations are compared and discussed with respect to the pure carbon slit-shaped pore topology which is our reference system [3].

## 3. Adsorption Limits from Numerical Perspective: Pure Nanoporous Carbons

**Figure 1:**
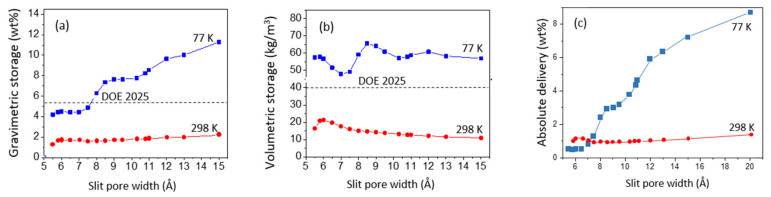
(**a**) The total gravimetric and (**b**) volumetric quantity of hydrogen stored in infinite carbon slit pores at T = 77 K and T = 298 K and the gas pressure of 100 bar; the dashed lines indicate the gravimetric and volumetric DOE goals for 2025 (55 gH_2_/kg of system, 40 gH_2_/L of system, respectively); (**c**) absolute hydrogen delivery during the adsorption–desorption cycle between 1 and 50 bar, as a function of pore width.

As the intermolecular H_2_–H_2_ interactions are relatively weak, the isotherms of H_2_ adsorption in carbon slit pores can be approximated by the Langmuir model, meaning that the adsorption mechanism does not involve specific features of gas or sorbent. However, the interplay between the energy of adsorption in pores of nanometric size and the thermal energy of the adsorbed gas leads to a non-trivial relation between total amount stored and pore volume and shape. For example, the storage capacity is not a linear function of the pore width (Figure 1a,b).

Moreover, the results presented on Figure 1a,b show that it is impossible to meet DOE goals for hydrogen storage by physisorption in nanoporous carbon at room temperature, regardless of the size of the pore. In fact, at high temperature, increasing pore volume does not lead to higher gas uptake: the thermal fluctuations prevent the stabilization of the subsequent layers on the contact layer, and in the middle of the large pores there is mainly non-absorbed (compressed) gas (Figure 4b in ref. [3]). The situation changes at cryogenic temperatures: the intermolecular H_2_–H_2_ interaction becomes non-negligible and the storage goal fixed by DOE can be achieved in carbons with pores larger than 0.9 nm. In pores larger than 1.15 nm, even a partial third layer (the middle one) between contact layers adsorbed on the pore walls can be stabilized.

For practical applications, the system’s ability to restore the adsorbed gas when the gas pressure decreases is as important as the storage capacity itself. The delivery is always low in narrow pores (of width smaller than 1 nm) (Figure 1c), independently of the temperature and the effective H_2_–H_2_ interaction, because of the strong cumulative interaction of adsorbed molecules with both pore walls. Therefore, although the presence of the narrow pores makes the amount of gas stored in excess of compressive storage larger, materials containing such ultra-micropores are not suitable for application because of low delivery efficacy. The delivery increases with the size of the pores; however, this is mainly due to the recovery of the gas compressed in the middle of the pore and not to the desorption of molecules from contact layers. High relative delivery is always possible at high temperature.

Summing up, standard activated nanoporous carbons with SSA ~2600 m^2^/g and mainly slit-like pores formed by locally parallel, large graphene fragments will not be able to reach the target hydrogen storage required for large scale (especially mobile) room temperature applications [3]. At room temperature, narrow pores (of width smaller than 1 nm) show low delivery efficacy, and the larger pores (of width larger than 1.2 nm) do not fulfill the volumetric storage requirements.

This constatation may change dramatically if the SSA of the sorbent or energy of hydrogen adsorption could be increased.

## 4. Towards Larger Specific Surface of Nanoporous Carbons—From Finite Size Pores to Open Carbon Frameworks

The simplest way to increase the storage capacity of a sorbent is to increase its accessible surface [24,25]. This can be achieved by constructing extended 3D porous networks from finite size structural building blocks. This strategy has been successfully applied to prepare metal and covalent organic frameworks (MOFs and COFs) [26,27,28,29], and porous aromatic frameworks (PAFs) [30] consisting of cage-like polyphenylene units. Theoretically, such structures can show a specific surface as large as 20,000 m^2^/g [31]; however, such highly porous materials have not yet been prepared. 

The same concept of the formation of extended porous structures by connecting small building blocks may also be applied to prepare highly porous all-carbon structures. An extensive review of constitutive models of porous carbons, using elementary building structures of finite size (aromatic rings or graphene fragments) was given by Palmer and Gubbins [32]. It was estimated [33] that a hypothetical nanoporous carbon built of small graphene fragments may have a specific surface 2–3 times larger than an infinite graphene plane. Model activated carbons, based on assemblies of graphitic micro-crystallites containing from 56 to 212 carbon atoms, have been proposed by Kaneko et al. [34]. The SSA of such graphene fragments increases when the amount of carbon they contain decreases. The graphitic fragment containing 56 carbon atoms has a specific surface of 5800 m^2^/g; this value increases to 7745 bm^2^/g for a hypothetical adsorbent build from isolated benzene molecules [31].

**Figure 2:**
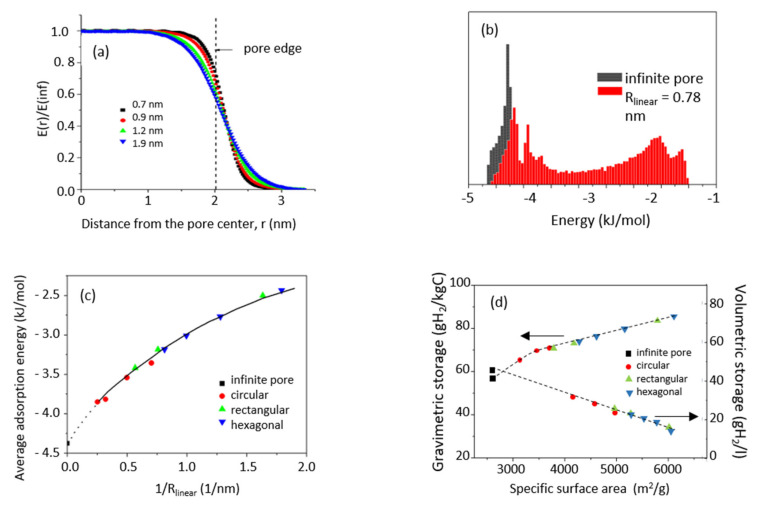
(**a**) Variation of the potential energy of a H_2_ molecule adsorbed in the mirror plane of a pore delimited by circular walls of the radius of 2 nm for four pore widths; (**b**) distribution of hydrogen adsorption energy on circular graphene fragment of the radius of 0.78 nm. For comparison the distribution of adsorption energy on infinite graphene surface is also shown (in black); (**c**) variation of average H_2_ adsorption energy in a function of graphene fragment size and shape: R_linear_ = (S_fragment_/π)^1/2^; (**d**) gravimetric and volumetric storage capacity vs. specific surface area of the model pore at the width of 1.5 nm, at *p* = 100 bar.

In addition to introducing additional surface area accessible to adsorption at the pore edges, fragmentation of the pore wall causes dramatic changes of distribution of adsorption energy in the sorbent. In finite pores, this distribution becomes strongly heterogeneous. The energy of adsorption (absolute value) is the largest in the volume around the axis connecting centers of the pore walls, and progressively decreases approaching pore edge (Figure 2a). Most importantly, a significant contribution of energies below 2 kJ/mol originating from outside the pore limits appears (Figure 2b). This contribution comes from the adsorption sites located at the pore wall edges, therefore it is totally absent in the infinite pore model. Although the energy of hydrogen adsorption on these sites is relatively low (less than half of the energy of adsorption on the infinite graphene wall), and the average H_2_ adsorption energy on the fragmented surface decreases when the fragment size decreases (Figure 2c), their large number may provide an important contribution to the total hydrogen uptake in porous carbons built from scaffolds of nanometric size.

It is important to remember that both gravimetric and volumetric storage are independent of the pore wall shape (Figure 2d). The gravimetric storage increases when the total adsorption surface increases (when the size of the nano-fragments decreases), and the fraction of the surface at the pore edges becomes an important part of the total surface available for adsorption [8]. In consequence gravimetric storage is always larger in finite pores than in infinite slit-like pores of the same width. On the other hand, fragmentation of the adsorbent wall makes, in general, the volumetric storage lower. This happens because the contribution of the adsorption in the volume around the edges (containing molecules adsorbed at lower density than inside the pore) to the total adsorption becomes non-negligible when the size of the pore wall is in the nanometric range. In consequence, volumetric storage in the limited size slit-like pores will always be smaller than in infinite ones of the same width.

Summing up, the nanoporous carbons formed from small graphene-based building units have, as expected, larger specific surface area, but the additional ‘edge’ surface adsorbs hydrogen with energy lower than an infinite graphene layer. This competition between increasing surface and decreasing average adsorption energy is the key issue limiting hydrogen storage in many ‘open’ structures, including MOFs, COFs, and PAFs. A wise choice of nanoporous carbon building elements which will balance these two tendencies is then necessary. 

**Figure 3:**
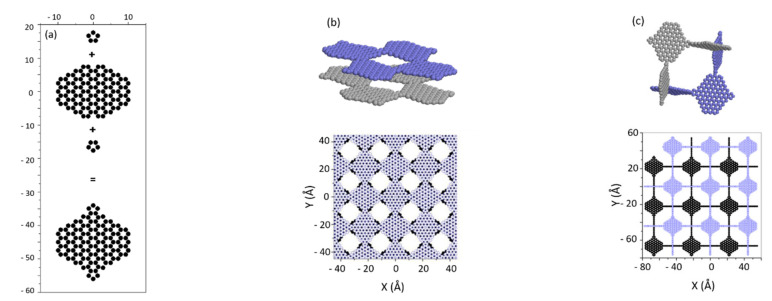
(**a**) Hypothetical polyaromatic molecule used to construct OCFs: half of its specific surface derives from surface of molecule edges; (**b**) 3D slit-like structure (SSA ~3800 m^2^/g) built from supermolecules shown in (**a**); (**c**) 3D orthorhombic structure (SSA ~4400 m^2^/g) built from supermolecules shown in (**a**).

Following this conclusion, we have proposed a hypothetical supramolecule consisting of 43 rings (116 carbon atoms, Figure 3a) as an elementary building unit of a new class of nanoporous carbons (Open Carbon Frameworks, OCFs) [6]. Its specific surface is ~4600 m^2^/g; half of that value comes from the surface edges. Using this supramolecule, two hypothetical periodic 3D nanoporous structures have been designed. The first (Figure 3b) contains four supramolecules in the unit cell (a = b = 4.432 nm) and has slit-type geometry. The second, orthorhombic structure has a cubic cell (a = b = c = 4.432 nm) with six supramolecules (Figure 3c).

**Figure 4:**
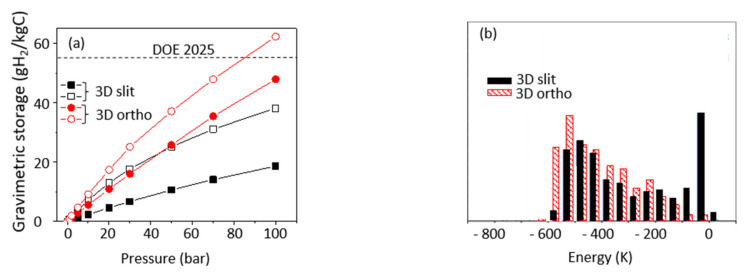
(**a**) Isotherms of total hydrogen adsorption in 3D slit-like and orthorhombic structures from Figure 3b,c. Open symbols refer to interaction model two times stronger that H_2_–C interaction; (**b**) distribution of adsorption energy in 3D slit-like and orthorhombic structures.

Both structures show impressive hydrogen storage capacity even at room temperature (~15 g/kg for silt-like structure and 45 g/kg for orthorhombic (Figure 4a, closed symbols). This performance results from the very ‘open structures’ topologies: gas adsorption at the (large) surface samples’ edges compensates for the decrease of the adsorption inside the pores (between pore walls) caused by the decrease of average adsorption energy in the systems (Figure 4b). In consequence, the proposed OCFs perform better than COFs [28] and PAH [30] materials, despite much smaller surface accessible for adsorption (3800–4200 m^2^/g for OCFs versus ~7100 m^2^/g for PAH-302 [30]). The hydrogen storage would be further improved if the strength of interaction of hydrogen molecules with the porous structure could be increased. We estimated that OCF could reach the applicative gravimetric DOE goals if their adsorption energy doubled (Figure 4a, open symbols). So, the challenge now is to find ways to make the interaction between hydrogen molecule and pore wall in real sorbents stronger.

## 5. Increasing Energy of Adsorption: Boron and Beryllium-Doped Carbons

**Figure 5:**
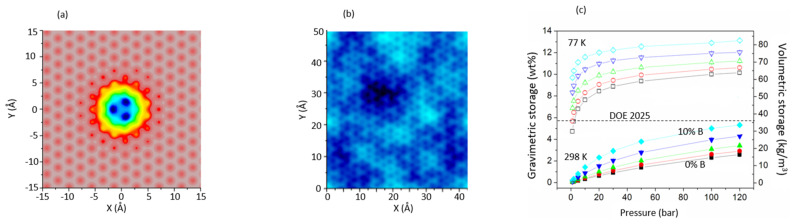
(**a**) Energy landscape for H_2_ adsorption over graphene layer with central carbon atom substituted by boron; (**b**) energy landscape for H_2_ adsorption over graphene layer containing 10% of substitutional boron atoms: the substitution is made at random, respecting the separation of two carbon sites between each pair of boron atoms; (**c**) gravimetric (left axis) and volumetric (right axis) storage capacity of boron substituted infinite slit pores as a function of H_2_ pressure, for different B:C substitution ratios.

The increase of the strength of hydrogen adsorption in carbon sorbents can be achieved by chemical modification of the pores’ surface. High storage capacities have been observed for alkali (Li, K) doped carbons [35]. Although some of these experimental results remain controversial, ab initio calculations show that Li doping can actually increase the adsorption energy by a factor of three [36]. Another group of materials considered as suitable materials for hydrogen storage are carbon structures partially substituted by boron. Boron acts as a p-dopant, introduces electron deficiency in the graphene layer, and increases the surface polarizability [37,38]. It was also suggested that a partial charge transfer occurs between the occupied H_2_ s orbital and the empty p_z_ orbital of carbon, leading to increase of interaction (adsorption energy) between H_2_ and the B-substituted carbon surface [39,40,41]. Following these conclusions, we performed ab initio calculations to obtain the energy of interaction between H_2_ and boron atoms substituted in graphene layer, modeled by pyrene. We showed that, despite the fact that the B–C bond is slightly longer than the C–C bond (1.59 Å vs. 1.42 Å), the optimized boron substituted surface preserves the planar structure of the carbon layer [12]. The minimum energy of H_2_ interaction with boron center E(B-H_2_) = −7.8 kJ/mol (at the optimized B–H_2_ distance of 3.12 Å) is only 50% larger than H_2_ interaction with graphene, but the H_2_ interaction with C_α_ (carbon directly connected to boron) is also modified (E = −5.6 kJ/mol, at the distance of 3.24 Å). These ab initio energies were parametrized to describe interaction energy between H_2_ and the boron substituted pore wall [12] (Figure 5a), for substitution ratios varying between 1% and 10% (Figure 5b). We showed that substitutional boron contributes to a dramatic strengthening of the adsorption energy over centers of neighboring hexagons. The modification of energy landscape extends far beyond C_β_, up to ~7Å from substituted boron atom (Figure 5a). A substitution ratio of 10% causes significant overlap of regions with modified energy, and the average adsorption energy increases, up to ~11.6 kJ/mol. This is more than double the energy of adsorption over a graphene layer. In consequence, the gravimetric storage capacity in the substituted infinite slit pores increases, and for 10% of substitution, at room temperature and H_2_ pressure of 100 bar, it approaches the DOE application target for 2025 (Figure 5c). It must, however, be recalled that the target requirements are formulated for the entire storing system, whereas our results address the material storage capacity only.

The wall heterogeneity and the presence of strongly adsorbing sites introduced by boron doping reduces by half the reversibility of H_2_ adsorption at low temperature (from ~53% for pure caron nanopores to ~25% in the same structures substituted by 10% of boron). However, the hydrogen delivery (in a complete adsorption/desorption cycle between 1 bar and 100 bars) is almost complete at room temperature (~97%), and does not depend on substitution ratio. Our calculations show that, at least theoretically, boron doped carbons, with adequately engineered pore wall structure, are the best candidates for practical use as hydrogen sorbents. 

There are very few attempts at experimental verification of numerical predictions for energy of hydrogen adsorption in boron-substituted carbons reported in the literature. The most extensive experimental studies of boron containing carbons have been published in a technical Report by the US Department of Energy (DOI) [40]. The report indicated that an energy of adsorption of 9.6 kJ/mol has been measured in carbon powder samples containing 1.7 wt% of boron; however, the nature of boron presence in the sample (substitution, doping or intercalation between graphitic structures) has not been determined. The report concluded that the high energy adsorption sites (up to 9.6 kJ/mol) represented a relatively small part of the available adsorption sites.

We proposed a totally new approach to introduce boron atoms into the carbon structures, in situ, as they grow in plasma produced by the electric arc discharge between graphite electrodes. The arc discharge method of synthetizing carbon scaffolds was usually applied to synthetize carbon allotropes (fullerenes and nanotubes). It can also be used to prepare substituted carbon structures containing heteroatoms [42,43]. To introduce boron atoms into plasma created between graphite electrodes, we have drilled a cylindrical hole into one of them (anode) and filled this with boron powder mixed with powdered graphite. By varying the boron to carbon ratio, the boron content in the final soot issued from the discharge could be varied in a quasi-continuous fashion between 0 and 25% (the upper limit resulting from the anode geometry). The details of the experimental protocol are described in the Supplementary Materials (Figures S1 and S2).

**Figure 6:**
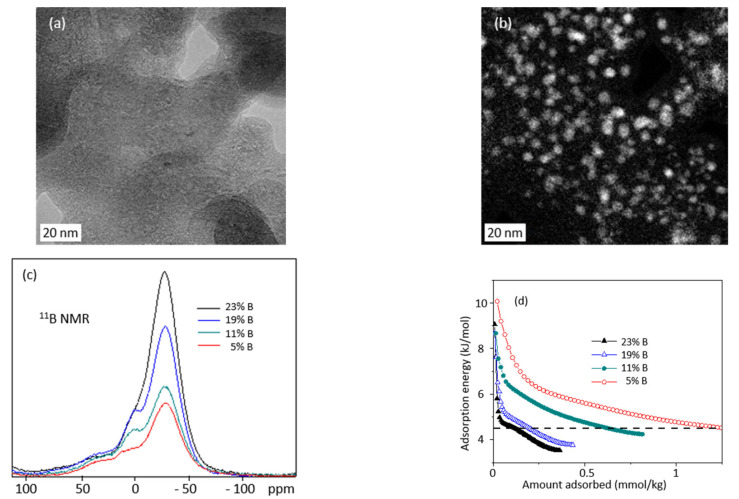
(**a**) HR TEM and (**b**) EELS images of the same fragment of sample containing 23 wt% of boron; (**c**) ^11^B HR spectrum of samples containing 5, 11, 19, and 23 wt% of boron. The ^11^B chemical shifts were referenced to H_3_BO_3_ 0.1 M solution; the MAS frequency of 10 kHz was used; (**d**) hydrogen adsorption energy in a function of gravimetric storage calculated from a pair of isotherms at 77 and 87 K (horizontal dashed line indicates 4.5 kJ/mol level typical for pure activated carbons).

The morphology and composition of materials fabricated using arc-discharge technique is strongly heterogeneous. Both amorphous and graphitized structures are produced (Figure 6a). The boron atoms form nanoclusters of variable size, uniformly dispersed in the whole sample (Figure 6a,b). The ^11^B (Figure 6c) and ^13^C HR NMR measurements confirm the presence of homonuclear B–B bonds (at the position typical for boron-carbide type structures), and of B–C bonds; their quantity grows when the boron content in the sample increases. 

The as-prepared samples have low porosity and small surface area (of the order of 200 m^2^/g) and require activation. The procedure, consisting of heating the samples in air at T = 400 °C during 4 h, increases the surface area of pure carbon samples by a factor of four. The process efficacy dramatically decreases when the boron content in the samples increases. In consequence the measured amount of hydrogen adsorbed in the samples is small, although sufficient to estimate the hydrogen adsorption energy from two isotherms of adsorption, measured at T = 77 K and T = 87 K (from Clausius-Clapeyron relation and assuming a Langmuir adsorption model).

The maximal adsorption energies, E_a_ ~9 kJ/mol (Figure 6d), estimated at the limit of zero H_2_ pressure, are in very good agreement with our previous numerical predictions of E_a_ ~10 kJ/mol. In general, the higher the boron concentration, the lower the number of high energy adsorption sites (Figure 6d). As the samples are heterogeneous, the adsorption energy always decreases with hydrogen loading: at low loadings it first drops very quickly down to about 5 kJ/mol, then continues to decrease more steadily, and stabilizes at ~3.5 kJ/mol for high hydrogen uptakes. This suggests that, according to our numerical analysis, the pore walls are very defected or fragmented, thus exhibiting the (averaged) adsorption energy even lower than 4.5 kJ/mol typically observed in activated carbons. For low substitution ratio (5% and 11%) the adsorption energy decreases more slowly with coverage which suggests more uniform distribution of the high energy sites, and consequently more extended modification of the energy landscape, not limited to a small number of substitution sites.

The experimentally observed increase (doubling) of energy of hydrogen adsorption in boron-substituted carbons (with respect to pristine activated carbons) constitutes a proof of concept that high adsorption energy is possible to attain in carbon-based nanoporous sorbents. 

A substitution of carbon atoms by beryllium is one of the least studied modifications of carbon adsorbents. However, some organo-beryllium compounds were proposed as promising hydrogen sorbents [39,44,45,46]. Beryllium can be introduced into the graphene sp^2^ network either as a single atom substitution (C/Be) or as a beryllium dimer (C/Be_2_). We have analyzed changes in the energy of hydrogen binding towards Be-substituted surfaces taking ovalene C_32_H_14_ as a model of graphene network and a starting/reference point for both types of substitution (for computational details, see [47]).

In general, if the C/Be substitution exists as an isolated site, the planarity of the system is perturbed, but the energy of hydrogen adsorption remains similar to that of pure graphene. This increases with the number of substituted sites, and reaches the value of 12.8–13.7 kJ/mol for C_5_Be-type structure (two substitutional Be atoms are separated by three carbon atoms). This value could be interesting from the applications point of view; however, the possibility of synthesis and stabilization of OCF structures based on such Be-substituted ovalene units has to be proven. Additionally, not all topologies of substituted surfaces are equally suitable for practical applications. For example, if two adjacent carbons in ovalene are substituted with Be, the hydrogen binding energy reaches a value of 46.5–49.5 kJ/mol, and reversible adsorption becomes impossible.

**Figure 7:**
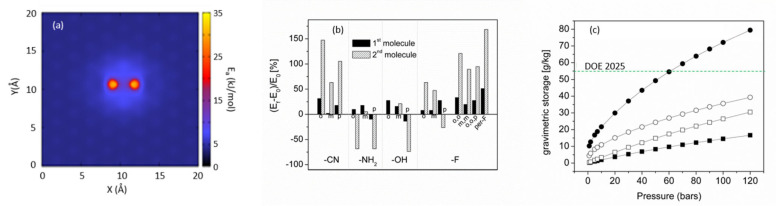
(**a**) Energetic landscape of infinite graphene surface substituted by two Be_2_ dimers separated by two carbon atoms; (**b**) variation (%) of energy of H_2_ adsorption over the Be_2_ dimer in functionalized Be_2_-benzene. E_0_ is the binding energy of H_2_ adsorbed on non-functionalized Be_2_-benzene: E_0_(1st H_2_) = 21 kJ/mol, E_0_(2nd H_2_) = 8 kJ/mol, (o)—ortho, (m)—meta, (p)—para position of the functional group with respect to Be_2_ dimer; (per-F) stands for per-fluorinated Be2-benzene; (**c**) hypothetical RT storage capacity in porous carbon with SSA = 5000 m^2^/g and the pore width of 1.1 nm: open squares-all carbon structure, open circles—25% of carbon sites substituted by Be_2_ dimers, close circles—25% of carbon sites substituted by Be_2_ dimers, 25% showing enhanced adsorption energy due to functionalization of pore edges; black squares—the RT storage capacity in infinite graphene-based slit-shaped pores of the same width.

Substitution of a single carbon atom by the Be dimer does not perturb the host graphene structure. The center of the dimer stays within the graphene plane, and the Be atoms (separated by 1.7 A) on the axis perpendicular to it. Each Be dimer can bind two molecules of hydrogen. The adsorption energy of the first molecule is about ~23 kJ/mol, close to the perfect value suggested for the optimal hydrogen sorbent [25]. The second molecule is adsorbed with lower energy, ~8 kJ/mol, but still nearly 50% higher compared to the pure graphene. The energetic modification of the potential energy remains localized on the substitution sites (Figure 7a): the adsorption energy on C_α_ carbons is almost the same as on carbons in graphene. In consequence, for a C/Be_2_ substitution ratio of 25%, the hydrogen uptake in infinite pores doubles with respect to carbon analogue (25 wt% at RT and a pressure of 120 bar), but remains below the application target value.

As the Be_2_-induced perturbation of the carbon configuration is very limited, further engineering of Be_2_-C_n_ composition and configuration remains possible. In particular, an addition of electron withdrawing groups is expected to decrease the electron density on Be atoms, therefore strengthening H_2_ adsorption energy on the Be dimer. The final binding energy depends on the chemical nature of functional groups, substitution topography, and the size of the substituted/functionalized graphene fragments [13]. Between four analyzed light weight and low volume electronegative functions (–CN, –NH_2_, –OH and –F), the cyano and fluorine groups have the largest impact on binding energy that increases by 5%–50% for the first adsorbed H_2_ molecule, and by 60%–150% for the second [13] (Figure 7b). Therefore, by varying the Be:C substitution ratio, the energy of hydrogen adsorption can be modulated in a wide range of values [13]. The partial (25%) functionalization of pore edges increases the gravimetric storage of Be_2_-substituted carbon, and even the ultimate (system) DOE storage target can be reached at pressure as low as 60 bar (Figure 7c). Although nanoporous organo-beryllium compounds are hardly applicable at the large scale because of their toxicity, our result puts into another perspective the directions to take in further experimental search for room temperature efficient hydrogen sorbents.

## 6. Discussion

In this review we have emphasized that the nanoporous carbons are one of the most intensively studied sorbents and very attractive materials for hydrogen storage by physisorption. In particular, many research groups have intensively looked for such derived materials that could reach the hydrogen storage capacity goals defined by the US Department of Energy (DOE) for mobile applications. This issue has been widely studied and discussed in many experimental and theoretical papers [48,49,50,51,52,53,54,55,56,57,58,59,60,61,62,63,64,65,66,67]. Within the conducted research, the experimental findings always played a critical role as they represent the ultimate verification of scientific ideas. In the case of hydrogen storage by physisorption, more than 20 years of intensive experimental effort led to the conclusion that pure-carbon nanostructured sorbents containing locally slit-like pores all fall short of the DOE targets. This is mainly due to the relatively low energy of adsorption of hydrogen on carbonaceous substrates, which is in the range of 3–6 kJ/mol. It is now clear that the development of more efficient sorbents requires significant chemical and structural modifications of currently known structures. In this quest numerical modeling has a leading role to play, as it allows for time-efficient, almost no-cost design of new materials with predesigned properties.

A careful examination of the extensive numerical database produced by Monte Carlo simulations of H_2_ adsorption in nanoporous carbons [4,5,6,7,8,9,10,11,12,13,14,48] allowed us to propose a simple formula allowing the calculation of the expected material gravimetric capacity (*G*, in g/kg) at room temperature in a function of material specific surface (*S*, in m^2^) and its average energy of hydrogen adsorption (*E*, in kJ/mol):

(1) $$G=f\times \left(S/1000\right)\times E$$

The coefficient *f* depends on system topology, mainly on the pores’ width [15].

**Figure 8:**
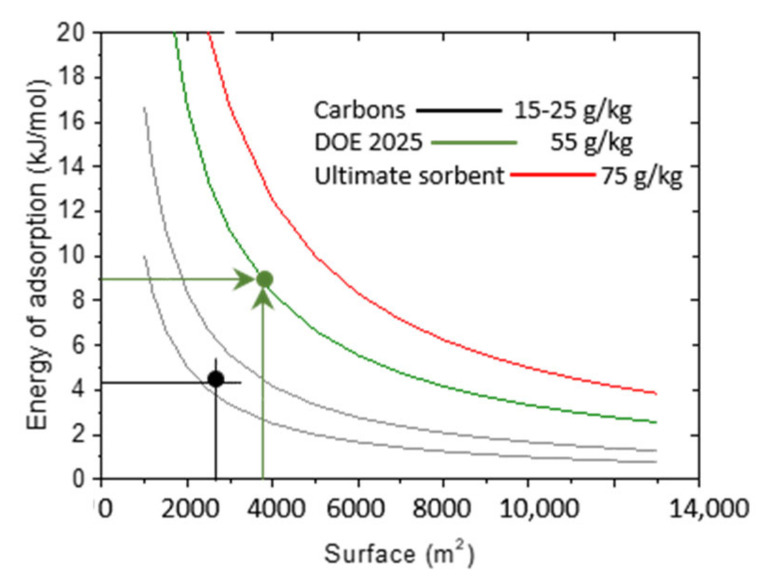
Iso-weight weight-capacity curves (G, in g of H_2_ per kg of sorbent) for G values typical of high surface activated carbons and the DOE ultimate goal. The intersections of the lines with G curves indicate the required surfaces (horizontal lines) and average binding energy (vertical lines) to achieve different storage capacities.

This relation allowed us to draw iso-weight-capacity curves (constant *G*) indicating the pairs of (*S*, *E*) values that are simultaneously needed to reach a given hydrogen storage capacity G (Figure 8). It also allows estimation, for required capacity, of what the required energy of adsorption should be if the total system surface (SSA) is known. 

The numerical results allow us to arrive at conclusions about the past numerical and experimental efforts to design and synthesize an optimal hydrogen sorbent. Most of such attempts have focused on improving only one of the material parameters (adsorption surface or adsorption energy), keeping the other constant. Such approaches can lead to success (reaching the applicative target storage) only when the second characteristic is significantly improved (specific surface larger than 10,000 m^2^/g or adsorption energy stronger than 20 kJ/mol). Simultaneous optimization of both parameters may be easier to achieve.

In the case of boron substituted carbons synthesized within the present study, the experimental energy of adsorption is of the order of 9 kJ/mol. Application of the formula above shows that, to arrive at a hydrogen capacity of 55 g/kg (DOE target for 2025, green line in Figure 8), the system specific surface has to be of the order of ~4000 m^2^/g (green horizontal arrow in Figure 8.) If the required capacity should reach 75 g/kg, the necessary surface should be engineered to attain 6000 m^2^/g.

Other possibilities for hydrogen storage (chemical storage in metal hydrides, or hydrogen-reach compound (mostly fossil fuel-based, such as methane), formation of Kubas complexes, chemical storage assisted by spillover of H_2_ molecules by Pt or Pd) have also been considered and described in the literature. An extended list of the storage methods and the storing materials are given in recent reviews [68,69]. However, both the storage and the release of hydrogen in these cases require high energy input, therefore lowering the energetic efficiency of such processes.

## 7. Conclusions

The major result emerging from the current state-of-the -art studies of hydrogen storage in nanoporous carbons is the numerical determination of the properties of a hypothetical carbon-based porous system, which would be able to adsorb enough hydrogen for mobile applications at industrial scale. We have also shown that a simple experimental path to achieve these parameters exists, although scaling this up may constitute a substantial technological challenge. 

The numerical results show that chemically modified, carbon-based sorbents with improved but reasonable surface area (4000–5000 m^2^/g) and larger adsorption energy (8–10 kJ/mol) become appealing hydrogen sorbents. Our preliminary experimental results show that such materials may be prepared using simple arc discharge between graphite electrodes stuffed with heteroatom-containing compounds. We also showed numerically that the adsorption energy may be additionally modulated by functionalizing already substituted carbon surfaces with strong electronegative groups. Next, an intensive research effort is required to optimize the synthesis and the activation procedures, to prepare porous structures with higher specific surface and more uniform distribution of the high energy adsorption sites. Our results give strong justification for the continuation of such research.

The present study focuses only on physical features of hydrogen storage by physisorption in carbon-based materials, and does not analyze the economic aspects of such a process. However, some facts seem to be evident. Nanoporous carbons can be prepared today from almost any organic waste (for example corncobs [63,64], nut shells [65], rice husk [66], fruits bunch [67], etc.) at relatively low cost, resulting from raw material carbonization and carbon activation with simple chemicals (steam, KOH, or NaOH) [7]), contributing in this way to sustainable gestion of society waist. The adsorption of an exploitable quantity of hydrogen can be achieved at relatively low pressure (under 120–150 bar), which decrease the cost of its compression, even if the heat released during the gas adsorption is not recovered. At the same time the recovery of the stored gas a priori does not require energy expense: a simple release of pressure is sufficient, and the decrease of temperature of the emptying tank can be, in many cases, compensated for by the heat exchange with the surrounding atmosphere.

## Data Availability

The data presented in this study are available on request from the corresponding author.

## Supplementary Materials

The following are available online at https://www.mdpi.com/article/10.3390/nano11092173/s1. Figure S1. The electric arc discharge reactor for synthesis of carbon nanostructures: (a) general view of the reactor; (b) schematic representation of the reactor cross-section. Figure S2. Cross-section of the drilled graphite rod prepared to serve as anode containing a powder of non-carbon material to synthetize substituted nanoporous carbons. In the present study, the drilled whole was filled with boron.
