# Supplementary material for: Hydrogen Storage in Pure and Boron-Substituted Nanoporous Carbons—Numerical and Experimental Perspective
Source: Nanomaterials (Basel). 2021 Aug 25;11(9):2173. doi: 10.3390/nano11092173 (PMC8472433; doi:10.3390/nano11092173)
Supplement: Supplementary file 1 [file nanomaterials-11-02173-s001.zip › nanomaterials-1333052-supplementary.pdf]

Supplementary Materials

Article

Hydrogen storage in pure and boron-substituted nanoporous carbons -numerical and experimental perspective

Lucyna Firlej[1,2,4*], Bogdan Kuchta[3,4], Katarzyna Walczak[1],Catherine Journet-Gautier [5]

[1] Laboratoire Charles Coulomb, University of Montpellier-CNRS, Montpellier, France
[2] Department of Physics and Astronomy, University of Missouri, Columbia MO, USA
[3] Laboratoire Madirel, University Aix Marseille-CNRS, Marseille, France
[4] Faculty of Chemistry, Wroclaw University of Science and Technology, Wroclaw, Poland
[5] Laboratoire des Multimatériaux et Interfaces, University Claude Bernard-CNRS, Lyon, France
* Correspondence: lucyna.firlej@umontpellier.fr

1. Synthesis of unsubstituted nanoporous carbons.

The optimal conditions of graphite sublimation in controlled, inert gas atmosphere that are necessary to produce closed carbon structures (fullerenes and carbon nanotubes) were determined by Zahab[1] and Journet[2]. The same electrical parameters of synthesis were used in the present study. The voltage between graphite electrodes was set to 50 V. The intensity of the electric current between electrodes (100 A) was maintained constant during the sublimation process by regulating the distance between the stationary cathode and the anode that consumes itself during sublimation. The inert gas (helium) pressure in the reactor was maintained in the range between 660 and 1100 mbar (the limits of operation of the reactor, see Figure SI-1).

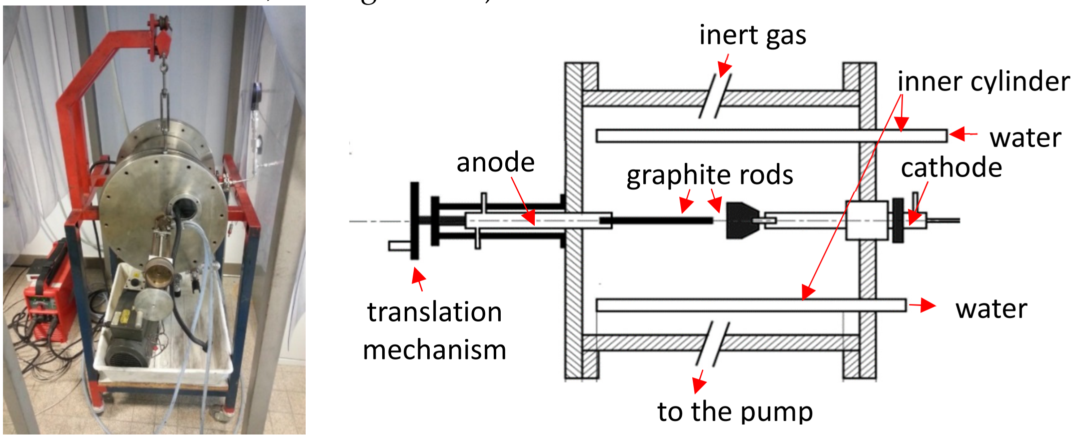

Figure S1. The electric arc discharge reactor for synthesis of carbon nanostructures: a) general view of the reactor; b) schematic representation of the reactor cross-section.

2. Synthesis of boron-substituted nanoporous carbons.

To synthesize the boron-substituted carbons we have used the same electric arc discharge reactor, in which the graphitic anode was replaced by the drilled one (see Figure SI-2), and filled with boron powder. The synthesis parameters (electric current, inert gas pressure) were the same as those already optimized for the synthesis of pure carbon samples. However, as boron is an insulator, it was more difficult to create or maintain a continuous electric arc discharge between electrodes. The optimal helium pressure to maintain an electrical connection between electrodes was found to be 800 mbar, even if simultaneously the difference of potential between the electrodes dropped from 50 V to 30 V. We collected and analysed only the soot part of the material. In such a way four boron-substituted carbon samples with boron concentrations of 23.3 wt.% B, 19.9 wt.% B, 11.3 wt.% B, 5.3 wt.% B were synthetized and characterized. The boron content has been estimated according to the formula

$$B(\%) = \frac{m_{ra} - m_{rb}}{m_{ra} - m_{rest}} \cdot 100\%$$

where m_{rb} is a mass of the rod before filling, m_{ra} is a mass of the rod after filling and m_{rest} is a mass of not sublimated anode, weighted after synthesis.

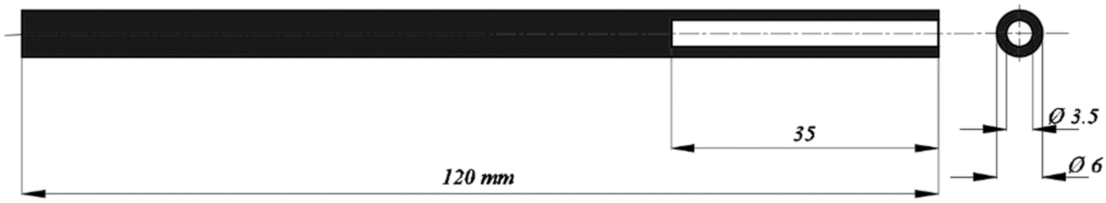

Figure S2. Cross-section of the drilled graphite rod prepared to serve as anode containing a powder of non-carbon material to synthetize substituted nanoporous carbons. In the present study, the drilled whole was filled with boron.

References:

1. Ahmed-Azmi Zahab, *Synthese, carcterization et etude physique des fullerenes C60 et C70 et de leurs derives*, PhD thesis, Montpellier, France, 1992
2. C. Journet, P. Bernier, *Production of carbon nanotubes*, Appl. Phys. A 67, 1-9 (1998)
